# Serial mediation effect of physical activity and sleep quality between dietary behaviour and depression symptoms: A nationwide cross-sectional survey

**DOI:** 10.7189/jogh.14.04081

**Published:** 2024-03-29

**Authors:** Lei Shi, Feiying He, Fangjing Wu, Yitao Ren, Man Xiong, Yibo Wu, Chichen Zhang

**Affiliations:** 1 School of Health Management, Guangzhou Medical University, Guangzhou, China; 2 School of Health Management, Southern Medical University, Guangzhou, China; 3 Entrepreneurship Academy of Southern Medical University, Guangzhou,China; 4 Department of Statistics, School of Arts and Sciences, Rutgers University, New Brunswick, New Jersey, USA; 5 School of Public Health, Peking University, Beijing, China; 6 Institute of Health Management, Southern Medical University, Guangzhou, China

## Abstract

**Background:**

Substantial studies have revealed the potential mechanisms underlying the link between dietary behaviour and depression symptoms. This study investigated the relationship between depression symptoms and dietary behaviour, physical activity, and sleep quality in a nationwide sample of Chinese residents.

**Methods:**

A total of 18 819 Chinese Residents completed the dietary behaviour, patient health questionnaire, international physical activity questionnaire, and Pittsburgh sleep quality index. We used the Hayes’ serial mediation model to investigate the correlation between the variables.

**Results:**

Among the participants, 85.5% were aged between 18 and 59, 41.2% were male, and 73.8% were urban residents. There is a negative correlation between dietary behaviour and physical activity (r = −0.038, *P* < 0.001), while there is a positive correlation with depression symptoms (r = 0.238, *P* < 0.001) and sleep quality (r = 0.115, *P* < 0.001). Additionally, depression shows a positive correlation with physical activity (r = 0.024, *P* < 0.001) and sleep quality (r = 0.298, *P* < 0.001), while there is a negative correlation between physical activity and sleep quality (r = −0.035, *P* < 0.001). Dietary behaviour was found to be connected with depression symptoms via three mediation pathways: (1) physical activity (B = −0.003, 95% confidence interval (CI) = −0.016, −0.007), (2) sleep quality (B = 0.034, 95% CI = 0.126, 0.164), and (3) physical activity and sleep quality (B = 0.001, 95% CI = 0.001, 0.003).

**Conclusions:**

These findings highlight the significance of psychological and physical factors in exploring the mechanisms through which dietary behaviour is related to depression symptoms. Overall, this study showed the important role of lifestyle factors in depression symptoms, suggesting that appropriate dietary behaviours, appropriate physical activity, and good sleep quality are necessary for the avoidance or improvement of depression symptoms.

Depressive disorder is among the top ten causes of the global burden of disease (2020) that can reduce quality of life and even lead to self-harm [1]. The prevalence of depression has increased over the past few decades. During the last three years, the number of people with major depression increased by 27.6% worldwide due to the coronavirus disease 2019 (COVID-19) pandemic [2,3]. Studies on the initial stages of the 2019 outbreak in China have shown a correlation between the epidemic and higher levels of anxiety and depression [4]. Despite its necessity, compulsory physical distancing interventions such as lockdowns and quarantine have been linked to social and physical isolation, resulting in unprecedented stress [5]. The stay-at-home order was associated with greater health anxiety, financial worry, and loneliness [6]. The ongoing pandemic has increased the severity of the global depression situation more than ever before.

COVID-19 also caused people to change their living habits [7]; the precaution of isolation modifies dietary behaviour and physical activity in a way that compromises health [8]. Negative dietary behaviours, such as unfavourable eating patterns or adverse habits, may cause depression and other affective disorders. Moreover, research has shown the two-way link of the correlation between dietary behaviour and depression, and that dietary behaviour can lead to depression through obesity [9], in turn, depression itself can affect eating habits, leading to overeating and weight gain [10]. Poor eating behaviours include for example skipping breakfast, ‘out-of-home’ (OH) food consumption, intake of sugary drinks, alcohol, and drinking inappropriate amounts of water. The frequency of breakfast consumption is associated with cognition, memory, motor and executive control, and other brain functions in all ages [11,12]. Increased breakfast omission is associated with an increased incidence of depressive symptom [13]. Besides omitting breakfast, people tend to prefer OH food to homemade or household food [14,15]. Out-of-home foods, such as takeaway, take-out, and fast foods, are known to contain excessive carbohydrates, fat, and salt, and a lower intake of fibre and vegetables [16,17], which are characteristic of unhealthy dietary patterns. A higher rate of OH food consumption implies poorer diet quality, which is associated with obesity [18], depression [19,20] and can even lead to cancer [21]. In addition to food consumption habits, drinking is equally important to human health. Sugar-sweetened beverages (SSBs) contribute the most to diet [22]. Sugar consumption is the primary risk factor for depression [23] Studies have also revealed a direct relationship between SSBs intake and depression [24,25]. Other drinks, such as alcohol and water, if consumed inappropriately, are also indicators of depression. According to Yang et al.’s study on a large Korean adult population, alcohol intake had a dose-response effect, but the association was not linear [26].

Furthermore, dietary behaviour may have indirect effects on depression; that is, a mediating effect of sleep quality may be linked to this association [27]. Massive evidence indicates that diet, including attached behaviours, can impact the sleep quality. Low intake of vegetables and high intake of confectionary and carbohydrates fit the characteristics of OH foods associated with poor sleep quality [28]. Consumption of SSBs and alcohol, omitting breakfast, and irregular eating had similar negative effects on sleep quality [29,30]. In contrast, sleep problems such as insomnia and lack of sleep duration are strong predictors of the emergence of depression [31,32]. Similarly, the association between dietary behaviour and depression may be mediated by physical activity. Favourable outcomes of physical activity on psychological health have been proposed [33], and a decreased frequency of physical activity, such as long sitting, could result in depression [34]. Recent studies have shown that the COVID-19 pandemic, along with its precautions, can lead to poor sleep quality and reduced physical activity [35–37]. Together with modifications in dietary behaviour due to the pandemic, people’s mental status has greatly been impacted. This study aimed to probe the interaction model of dietary behaviour, physical activity, sleep quality, and depression and determine their relationship mechanism.

Notwithstanding the abundant research findings on the association between each of the two variables of dietary behaviour, depression, sleep quality, and physical activity, the mediation or moderation effect of the variables was included in one model. However, existing literature does not fully consider multiple mediators [27]. Moreover, the majority of research concerning similar topics used a rather small sample size or focused on a specific crowd [38], making it difficult to draw relevant conclusions. Therefore, further explorations of the influencing factors need to be actualised, as depression will continue to be of interest. This study aims to shed light on innovative approaches for enhancing the mental health and living conditions of depression patients. First, the survey was conducted nationwide, including adults of all sexes and age groups in China, and the research focused on depression caused during the COVID-19 pandemic. Second, the study introduced OH food consumption, which has seen a significant increase in China, as reported in the 2023 Development Report on China's Sharing Economy. This trend is attributed to the country's unique social structure and composition.

The study aims to explore the intricate relationship between dietary behaviour and depression symptoms, specifically examining if sleep quality and physical activity act as mediators in this association and if there is a modulation effect of sleep quality and physical activity within this context. Consequently, three hypotheses were proposed (**Figure 1**): First, the study hypothesised that physical activity played a mediating role in linking dietary behaviour with depression symptoms (H1). Second, it was hypothesised that sleep quality served as a mediator within the relationship between dietary behaviour and depression symptoms(H2). Finally, it was hypothesised that the relationship between dietary behaviour and depression symptoms was sequentially mediated by both physical activity and sleep quality, indicating a series of mediating effects (H3).

**Figure 1:**
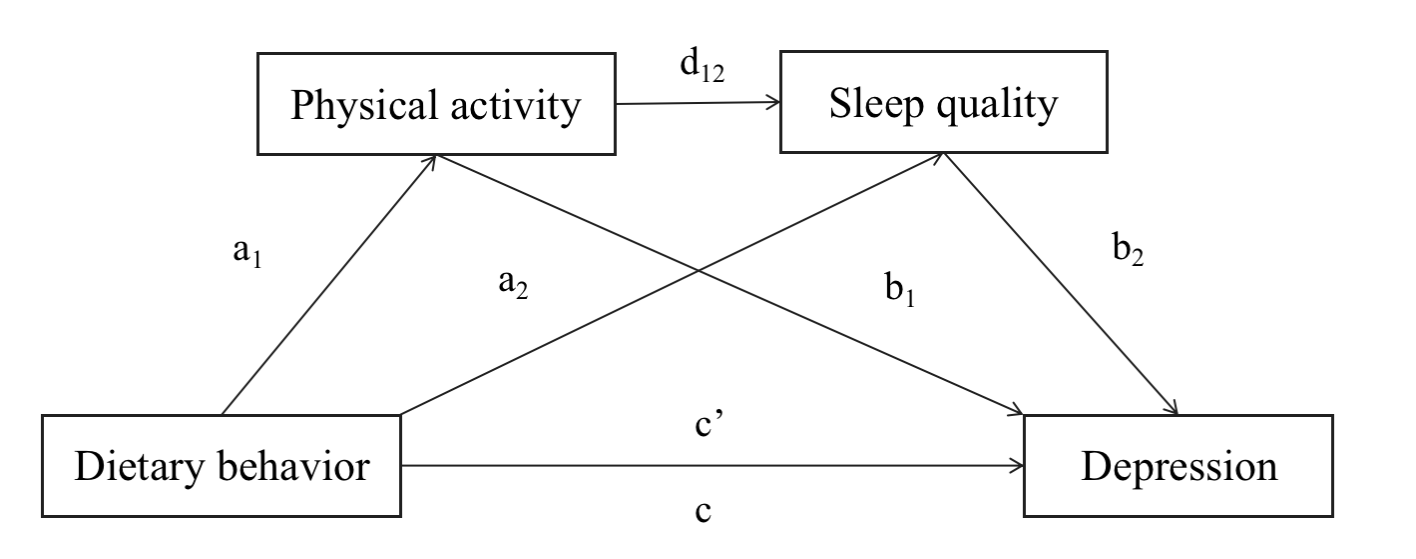
Hypothesised serial mediation model linking dietary behaviour and depression through physical activity and sleep quality as serial mediators. *a, b, c, c’, and d represent path coefficients.

## METHODS

### Survey sample and participants

The research utilised data from the Psychology and Behavior Investigation of Chinese Residents (PBICR), a comprehensive, cross-sectional survey designed to reflect the national demographic of China. Conducted between 20 June and 31 August 2022, the survey spanned 148 cities, 202 counties, and 309 villages/towns across 23 provinces, four municipalities, and five autonomous regions of mainland China, deliberately excluding Taiwan, Hong Kong, and the Macao Special Administrative Region. The selection of clusters adhered to the principle of equal probability, ensuring each region had an equal chance of being included, while individual respondents were chosen based on specific criteria. Eligible participants were those aged 18 years and above, who were permanent residents of mainland China and had not been abroad for more than one month within the past year. Exclusion criteria were established for individuals who were unconscious, encountered communication barriers, or exhibited cognitive impairments. Out of 30 505 initial participants, 28 674 met the criteria and completed the survey, leading to a high questionnaire response rate of 94.3%.

The questionnaire development process commenced with an exploratory phase, leveraging existing field knowledge and supplementing it with expert consultations and preliminary tests to enhance its effectiveness. The survey's sampling strategy was meticulously designed to enhance the representativeness of the study population. It combined stratified sampling, which ensured equal probability across different administrative divisions, with quota sampling, which allowed for non-equal probability from the community/village level down to individuals. The survey was administered through direct interaction or live video support, allowing investigators to effectively guide participants. Before participating, individuals were required to provide informed consent, having been fully informed about the study's objectives and the measures implemented to ensure anonymity, confidentiality, and the protection of their rights. The careful preparation and execution of this study underscored our commitment to ethical standards, as well as our aim to gather valuable insights from the 18 819 participants aged 18 and above.

### Measurements

#### Demographic variables

The collected demographic data included information on sex, age, marital status, education level, monthly family income, residence (urban/rural), quarantine status (positive/negative), community closure (positive/negative), smoking status, and chronic comorbidities. The study population was divided into two age groups: 18–60 years old and 60+ years old. Marital status was categorised as single, married/common-in-law, divorced/separated, or widowed. Education level was classified as primary school or lower, junior high school, senior high school, or college or higher. Monthly income was categorised as: less than 3500 Chinese yuan renminbi (CNY), 3500–5999 CNY, 6000–8999 CNY, 9000–12 000 CNY, and more than 12 000 CNY. Smoking status was divided into regular cigarette smokers and nonsmokers; electronic cigarettes were not considered. Chronic comorbidities were grouped into three categories according to the number of comorbidities: 0, 1, and >2.

#### Dietary behaviour

The study examined the impact of five factors on depression: alcohol consumption (coded as 0 = never drink, 1 = used to drink but don't now, 2 = didn't drink but do now, 3 = always drink), sugar-sweetened beverage consumption in the past week (0 = never, 1 = 1–3 cups/week, 2 = 4–6 cups/week, 3 = 7+ cups/week), daily drinking status (0 = <1200 ml, 1 = 1200–1499 ml, 2 = 1500–1699 ml, 3 = 1700–2099 ml, 4 = >2100 ml), overall OH food consumption (0 = never dine out, 1 = ≤1 time/week, 2 = 1–2 times/week, 3 = 3+ times/week), and breakfast omission (0 = never eat breakfast, 1 = 1–2 times/week, 2 = 3–4 times/week, 3 = 5–6 times/week, 4 = eat breakfast daily). Along with physical activity, sleep quality, and covariates, these factors were individually regressed on depression to yield coefficients representing their respective weights. The Weighted Linear Combination (WLC) method was used to construct a composite independent variable representing dietary behaviour based on these five factors. The five factors were regressed on depression as separate variables, along with physical activity, sleep quality, and covariates to obtain coefficients as their weights. The score of each factor was multiplied by its coefficient and then added up to constitute the independent variable, namely dietary behaviour, with a higher score indicating worse overall dietary behaviour.

#### Depression symptoms

Depressive symptoms were assessed using the self-administered 9-item depression module from the full Patient Health Questionnaire (PHQ), which has been applied to several populations [39–41]. The resulting scale for depression ranges from 0 to 27, with a higher total score representing higher risk or severity. A PHQ-9 score of greater than 10 exhibited 88.0% sensitivity and 88.0% specificity for serious depression. Studies have validated the PHQ-9 while screening for depression in the Chinese population [42]. In this study, the Cronbach's alpha coefficient for the PHQ was 0.859.

#### Physical activity

Physical activity was measured using the short form of the International Physical Activity Questionnaire (IPAQ) [43], which includes seven items. Based on the calculated weekly metabolic equivalents (MET) minutes, respondents were classified into three groups according to the IPAQ scoring protocol: (1) low active (<600 MET-h/week); (2) moderate active (≥600 MET-h/week); and (3) high active (≥3000 MET-h/week). The IPAQ has previously been shown to be credible across 12 countries and is valid for the Chinese population [44]. In this study, the Cronbach's alpha coefficient for the PHQ was 0.764.

#### Sleep quality

The brief version of the Pittsburgh Sleep Quality Index (B-PSQI) was used to assess sleep quality [45,46]. Only six of the original 18 questions were included in the brief version, which improved efficiency and applicability and reduced the impact of response bias. All items, including sleep duration, sleep latency, sleep efficiency, sleep disturbances, and subjective sleep quality, were scored from 0 to 3. Sleep efficiency was calculated by dividing sleep duration by the difference between the first two questions: ‘When have you usually gone to bed at night?’ and ‘When do you usually get up in the morning?’ The final score ranged from 0 to 21, with a higher score indicating poorer sleep. The B-PSQI is a helpful tool for evaluating sleep-related symptomatology in psychological disorders and is valid and reliable for testing poor Chinese sleepers, as demonstrated [47]. In this study, the Cronbach's alpha coefficient of the PHQ was 0.707.

### Data analysis

The analysis of depression differences across sociodemographic characteristics was performed using the Kruskal-Wallis test. Associations between all the study variables were determined using Spearman’s correlation. Statistically significant variables in a multi-variate analysis were included as covariates. Given the correlation found between the two mediating variables, physical activity and sleep quality, this study chose the serial mediation model to further explore how this relationship collectively influences the link between dietary behaviour and depression. To accomplish this, we conducted the analyses by using the PROCESS model, which was developed by Hayes [48]. The serial mediation model allowed us not only to examine how each mediating variable independently mediates the effect of dietary behaviour on depression, but also to analyse how these mediators interact to jointly mediate this relationship. This approach is a nonparametric resampling technique involving random and repeated subsampling and has greater statistical power than the traditional causal approach proposed by Baron and Kenny [49]. In this study, the independent variable, dependent variable, and two mediators were dietary behaviour (X), depression (Y), physical activity (M1), and sleep quality (M2).

Although it has been illustrated that mediation models are ideally examined using prospective data, theoretically-driven models of cross-sectional study testing indirect effects can also be appropriate [48]. Multiple mediation (PROCESS/Hayes Model 6) analyses were based on bootstrapping (5000 bootstrap samples) with 95% confidence intervals (CI). All analyses were conducted using the R version 4.2.0, with the significance level set at 0.05 (two-tailed).

### Common method biases

The same person completed the survey questionnaires; therefore, the problem of common method bias could not be eliminated. To control for common method bias effects, the procedures were standardised as much as possible, such as choosing a measurement instrument with high reliability and validity, responding anonymously, and using reverse scoring statements for scale items. Harman’s single-factor test was conducted to detect common method bias, which is widely used in common method bias testing [50,51].

## RESULTS

### Common method biases analyses

After the principal component analysis, six factors were extracted with eigenvalues greater than one. The first factor explained the common variation in all items of the research variables, which were created by common method biases and the relationships between the research variables. However, only 15.8% of the variance is explained by the first factor, which is far below the critical value of 40.0% [50]. Accordingly, the research concluded that common method bias was not a serious problem in this study.

### Preliminary analyses

The sociodemographic factors of the participants and the corresponding distributions of depression are shown in **Table 1**. A total of 18 819 participants aged 18 years and older were included in the study (85.5% between 18 and 59 years; 14.5% > 60 years). Among the participants, 41.2% were male and 58.8% were female. Of the participants, 73.8% has lived in urban areas for the past three months, and the other 26.2% lived in rural areas. The mode of income ranged from 2000–4000 per person per month (33.9%). The mean score for depression was 3.047 (standard deviation (SD) = 2.860). In the construction of the statistical model, only the covariates with *P*-values falling below the significance level of 0.05 were included.

**Table 1:** Sociodemographic characteristics of included participants in depression (n = 18 819)

Variables	n (%)	Depression (M ± SD)		*P*-value
Gender			43.129	<0.001
*Male*	7756 (41.2)	2.945 ± 2.941		
*Female*	11 063 (58.8)	3.119 ± 2.797		
Age			65.101	<0.001
*18–60*	16 088 (85.5)	3.113 ± 2.880		
*≥60*	2731 (14.5)	2.657 ± 2.693		
Education			52.257	<0.001
*No education experience*	692 (3.7)	2.893 ± 2.722		
*Secondary school or lower*	8004 (42.5)	2.912 ± 2.860		
*College or higher*	10 123 (53.8)	3.165 ± 2.860		
Income (CNY)			18.053	0.001
*<2000*	3420 (18.2)	3.242 ± 2.985		
*2001–4000*	6265 (33.3)	3.001 ± 2.833		
*4001–6000*	4496 (23.9)	2.946 ± 2.771		
*6001–12 000*	3279 (17.4)	3.066 ± 2.832		
*>12 000*	1359 (7.2)	3.058 ± 2.972		
Residence			2.791	0.095
*Rural*	13 897 (73.8)	3.023 ± 2.841		
*Urban*	4922 (26.2)	3.114 ± 2.905		

M – mean, Chinese yuan renminbi – CNY, SD – standard deviation

**Figure 2:**
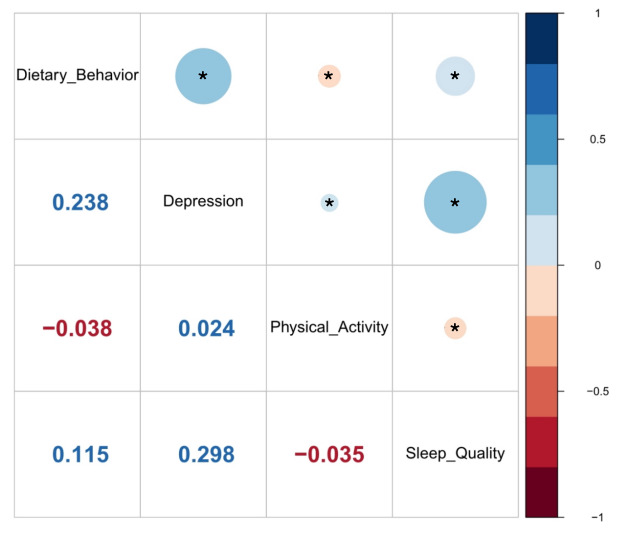
The Pearson correlation matrix of study variables. *P < 0.001.

The correlations of all four main variables are shown in **Figure 2**. Dietary behaviour was negatively correlated with physical activity (r = −0.038, *P* < 0.001), and was positively related with depression symptoms (r = 0.238, *P* < 0.001), and sleep quality (r = 0.115, *P* < 0.001). Depression symptoms correlated with physical activity (r = 0.024, *P* < 0.001) and sleep quality (r = 0.298, *P* < 0.001), respectively. The two mediators, physical activity and sleep quality, were negatively correlated (r = −0.035, *P* < 0.001).

### Multiple mediation analyses

**Figure 3:**
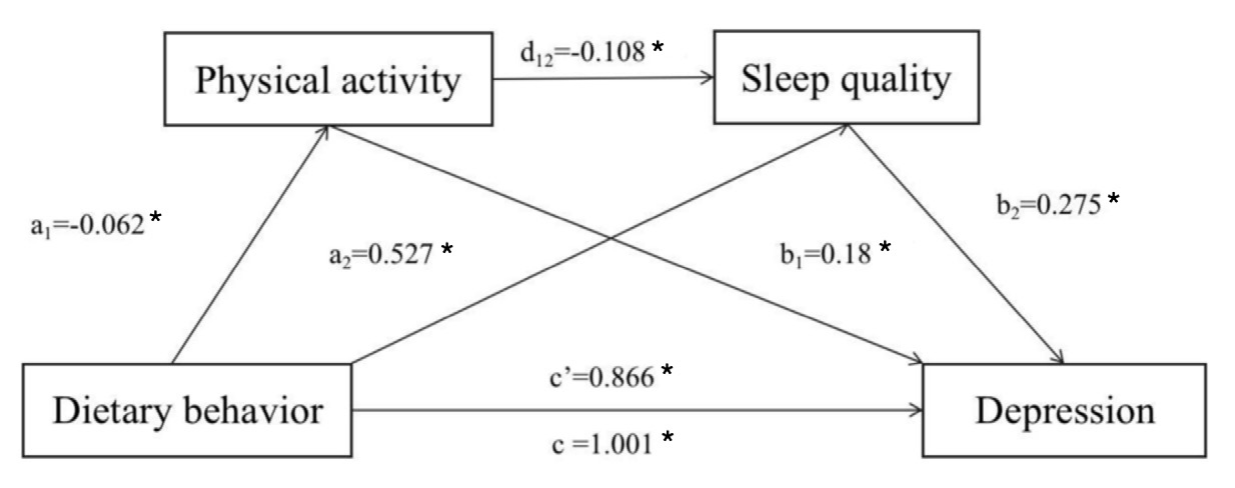
The serial multiple mediation role of physical activity and sleep quality in the relationship between dietary behaviour and depression. *P < 0.001.

Multiple mediation analyses were conducted to explore the mediating roles of physical activity and sleep quality in the relationship between dietary behaviour and depression symptoms. Dietary behaviour and depression symptoms were included as the independent variable and the dependent variable, respectively, in the model. Physical activity and sleep quality were included as two mediators. Control variables included gender, age, education, income, and residence. As shown in **Figure 3**, dietary behaviour exhibited a positive connection with depression symptoms (c = 1.001, *P* < 0.001). The model further revealed that dietary behaviour was negatively related with physical activity (a1 = −0.062, *P* < 0.001), and positively related with sleep quality (a2 = 0.527, *P* < 0.001). Both physical activity and sleep quality demonstrated significant correlations with depression symptoms (b1 = 0.180, *P* < 0.001; b2 = 0.275, *P* < 0.001). Notably, the two mediators, physical activity and sleep quality, were negatively correlated with each other (d12 = −0.108, *P* < 0.001).

According to the serial mediation model (**Table 2**), dietary behaviour was found to connected with depression symptoms via three mediation pathways: (1) physical activity (B = −0.003, 95% CI = −0.016, −0.007), (2) sleep quality (B = 0.034, 95% CI = 0.126, 0.164), and (3) physical activity and sleep quality (B = 0.001, 95% CI = 0.001, 0.003). The effect of dietary behaviour on depression remained significant with the two mediators added in the model (c’ = 0.865, *P* < 0.001).

**Table 2:** Direct and indirect effects of dietary behaviour (X) on depression (Y) through physical activity (M1) and sleep quality (M2) (n = 18 819)

Path	Estimate	Bootstrap SE	Beta	Bootstrap LLCI*	Bootstrap ULCI*
Total effect X→Y	1.001	0.032	0.238†	0.939	1.067
Direct effect X→Y	0.865	0.031	0.206†	0.806	0.931
Total indirect effect X→Y	0.136	0.010	0.032†	0.116	0.155
*Specific indirect effects*					
X→M1→Y	-0.011	0.002	0.003†	-0.016	-0.007
X→M2→Y	0.145	0.010	0.034†	0.126	0.164
X→M1→M2→Y	0.002	0.001	0.001†	0.001	0.003

CI – confidence interval, LLCI – lower limit of confidence interval, SE – standard error, ULCI – upper limit of confidence interval

*95% confidence interval.

†*P* < 0.001.

## DISCUSSION

The nationwide study explored the relationships of different variables with depression. Consistent with our hypotheses, the correlation analysis revealed a substantial pairwise association between dietary behaviour, physical activity, sleep quality, and depression symptoms. Dietary behaviour showed a robust correlation with depression symptoms in the regression model, and physical activity and sleep quality were correlated with depression symptoms. There is also a link between physical activity and sleep quality. The outcome of our study revealed two simple mediation effects of physical activity and sleep quality when they were set as mediators. That is dietary behaviour and depression symptoms can affect each other through physical or sleep quality. Our mediation model also revealed a serial mediation effect of physical activity and sleep quality on the relationship between dietary behaviour and depression symptoms, which indicated that the mutual effect of physical activity and sleep quality should also be considered when exploring the correlation between dietary behaviour and depression symptoms.

This study indicated that healthy dietary behaviour might diminish the risk of depression symptoms, consistent with Shafiei’s conclusion that better behaviours facilitate lower odds of major depression, contributing to better psychology [52,53]; whereas poor behaviours was significantly connect to the occurrence of depression syndrome. A high frequency of OH food consumption and breakfast omissions might be indicators of depression, echoing Sánchez-Villegas’s study [19]. Out-of-home food contains excessive carbohydrates, fat, and salt and less fiber and vegetables [16,17]. The Western dietary pattern is generally considered harmful to psychological health due to its high sugar, fat, and salt content, while the Mediterranean food pattern, comparably insipid and containing more fish, vegetables, and nuts are rather favourable diet patterns [54,55]. However, Hitomi's research was unable to confirm a definitive relationship between dietary patterns and the risk of postpartum depression [56]. This inconclusiveness may be attributed to the particular characteristics of the analysed sample. Inappropriate dining habits could be a trigger to mental unwellness. Omitting breakfast was considered to be a potential predictor of depression symptoms [18], and a significant correlation can be observed in those with a lower socioeconomic status [56,57]. In studies by Seguin and Janssen, adolescents' emotional issues were linked to skipping breakfast or having breakfast out of home [16,58]. For example, inappropriate consumption of liquids, including excessive or insufficient water intake, alcohol, and SSBs, has detrimental effects on the risk of depression symptoms. Among the five factors integrating dietary behaviour in this study, alcohol consumption had the most prominent relationship with depression symptoms. According to Gémes [59], when compared to non- or moderate alcohol consumption, hazardous alcohol consumption was linked to a higher risk of depression in Swedish individuals. Research on beverages, including soft drinks and juices, has demonstrated a direct effect with depression. SSBs with artificially added sugar have an even more severe impact [25,60]. Moreover, regular SSBs can lead to a later bedtime and worse sleep duration [29]. In contrast to consuming alcoholic and sugar-added beverages, consuming water is recommended for healthy hydration and contributes significantly to total water intake [61], which should be >1.8 l per 24 h [62]. Drinking less water daily could be a latent risk factor for depression, while more water intake was associated with more active exercise and greater nutrient consumption [63]. Based on the relationships examined above, an effective way to diminish risk of depression or improve depression syndrome should start by developing good dietary behaviours. This includes having regular breakfast and a better diet pattern, such as traditional or Mediterranean patterns with less consumption of SSBs and alcohol, increased water intake, and regular breakfast consumption.

The model was significant when physical activity was considered as a simple mediator, which supported Hypothesis 1. This study showed that compared with other indirect effect pathways, the sequential indirect effect of dietary behaviours on depression symptoms through physical activity had a greater influence. In line with Christofaro’s outcomes, a healthy diet, such as the Mediterranean diet instead of the Western diet, was followed by improved physical activity, particularly during the pandemic. Specifically, vegetables and fruits are favored components, while sweets and fried foods that can contain high OH foods and drinks are the opposite [34,64]. Conversely, higher energy expenditure leads to improved appetite, influencing dietary habits and creating a positive feedback loop. In addition, several papers covered and validated that physical activity productively ameliorates depression syndromes. Aligned with our results on the negative relationship between physical activity and depression symptoms, research has shown that engaging in regular physical activity is linked to a significantly lower likelihood of developing certain mental disorders, including depression, and appears to decrease the severity of co-occurring conditions [65]. Physical activity is also thought to improve depression in chronic kidney disease patients [66]. In some cases, physical activity can be used as a treatment, with a performance comparable to that of antidepressants [67]. Based on the relationships, the study found that negative dietary behaviour can cause depression symptoms through physical activity, whereas improved physical activity can diminish this link. However, it is important to note that research has also found that excessive exercise might increase the risk of depression symptoms, highlighting the need for a balanced approach to physical activity [68]. Hence, it is advisable to engage in physical activity of suitable duration and intensity.

Hypothesis 2 was also substantiated: sleep quality mediates the effects of dietary behaviour on depression symptoms. The relationship between diet and sleep quality has been investigated in several populations worldwide. Research implies that a healthy diet is a protective indicator of sleep quality, containing less sugar, beverages, and more fruits and vegetables [68], whereas an unhealthy diet has the opposite effect on sleep. Breakfast omission had manifested as influences on sleep in school-aged children [69]. The disturbance of alcohol on sleep quality is significant, whether in healthy people or in those with sleeping problems [70]. Next, the study investigated the effect of sleep quality on depression symptoms in several dimensions. One review clearly concluded that insomnia had a considerable effect on depression [32]. Another review summarised the impact of sleep duration on depression [71]. In particular, people with no affective disorder would have a 2-fold risk of developing depression symptoms if accompanied by insomnia compared to people with no sleep difficulties. All the studies indicated a negative correlation between sleep quality and depression symptoms. Our results revealed the mediating effect of sleep quality between dietary behaviour and depression symptoms, which means that not only poor sleep can trigger depression symptoms, but improper dietary behaviour could also lead to worse sleep quality and depression symptoms. The effects of dietary behaviour and sleep quality can be severe in terms of mental status. Consequently, attention should be paid to diet behaviour and sleep quality to maintain psychological health.

Although relatively subtle, the examination of sleep quality and physical activity as joint mediating variables in the association between dietary behaviour and depression symptoms in the serial model was statistically significant, as verified in our model. Specifically, dietary behaviour can be influenced by physical activity and sleep quality separately, and can also affect sleep quality through physical activity, thereby causing depression symptoms. Evidence for the direct and indirect effects of dietary behaviour on depression symptoms through mediating factors has been supported by previous study research [72,73]. One study explored the factors connected with life, including social support, sleep quality, health-related quality of life, and depression symptoms [38]. This suggests that depression symptoms can be indirectly affected by dietary habits through their effect on sleep quality. However, the effect of dietary behaviour and depression symptoms was not significant in their model, which might be because their sample size was less than 300, which was too small, or because the scale they used to represent dietary behaviour only covered diet ingredients. Although some research found that the relationship between dietary behaviour and depression symptoms could be due to habit schema or food intake that alters the functions of nerves or internal secretions, the specific mechanism of the multi-mediation effect on depression symptoms in this study remains unknown. In conclusion, the findings of this study provide a clear picture of the mediating pathways between dietary behaviour and depression symptoms. Dietary behaviour can cause depression symptoms through two mediators: physical activity and sleep quality. Furthermore, poor dietary habits inhibited physical activity, which interfered with worse sleep quality and combined with depression syndromes. This study explored the mediation effects between two variables that are of great importance at present, using comparably representative data from a nationwide survey. Based on the results of this study, mental health problems are notable, especially in children, adolescents, and older people. To maintain a better mental status, dietary behaviour, physical activity, and sleep quality are indispensable directions to consider. A 'healthy and wealthy' diet, along with good eating habits, is one way to stay fit. However, physical activity also plays a vital role in life. It can calibrate dietary behaviour and improve sleep quality, which requires sufficient sleep duration and appropriate bed timing.

To some extent, the findings of this study can be generalised beyond the Chinese population, given that the unhealthy dietary behaviours discussed in this research are not exclusive to the Chinese population. However, cultural disparities, social norms, and economic conditions limit its generalisability. Incorporating intercultural and global samples, particularly in the context of the mediation effect between dietary behaviour and depression symptoms, in future studies would expand the generalisability and universality of the findings.

### Limitations

This study has several limitations. First, our study was cross-sectional, limiting our ability to draw firm conclusions about causality between dietary behaviour and depression symptoms, physical activity, and sleep quality. Future longitudinal studies are recommended to clarify these relationships and establish the mediating roles of physical activity and sleep quality in the putative causal link between dietary behaviour and depression symptoms. Second, although this study incorporated two widely discussed predictors of depression symptoms – physical activity and sleep quality – as mediators, it could only account for a minor indirect correlation between dietary behaviour and depression symptoms. The link between dietary behaviour and depression symptoms was found to be considerably strong. However, the indirect effect is too intricate to be explained solely by these two mediators. To develop a more comprehensive and robust understanding of the underlying mechanisms, it is expected that additional variables will be incorporated into the model in future studies. Third, the research focused on specific unhealthy dietary preferences that have emerged recently. These preferences have the potential to give rise to a new national dietary mode with a profound impact. The questions that made up dietary behaviour in the study were novel and notable, yet too few to cover the complete aspects of diet and its dependence behaviour of an individual. Hence, more factors should be considered when integrating dietary behaviours. In addition, the nationwide data set based on the entire population expect stratification analyses to explore discrepancies in the mediation effect on the relationship between dietary behaviour and depression symptoms between different regions, ages, and genders. The serial mediation effect of the variables studied in this research was not tested to determine its persistence among specific subgroups of patients. Therefore, subgroup analyses could not be conducted to assess its robustness. Additionally, the prevalence of COVID-19 has fluctuated across different countries, and these situations continue to evolve due to varying environmental conditions and policies. Further studies are required for deeper understanding of the psychological status effect factors as more and more countries entered the ‘post-pandemic era’.

## CONCLUSIONS

This study provides empirical evidence for the pathways linking dietary behaviours to depression using a Chinese population sample. Depression symptoms is associated with dietary intake both directly and indirectly. Specifically, dietary behaviour increased the risk of depression symptoms via the mediating effect of inferior sleep quality, while improving depression symptoms through physical activity. Thus, it is conceivable that this link with dietary behaviour is partially due to a causal link between physical activity and sleep quality. This study highlights the significance of psychological and physical factors in exploring the mechanisms through which dietary behaviour is related to depression symptoms. Depression interventions aimed at improving mental health can benefit from strategies such as guidance on proper dietary behaviour, enhanced exercise frequency and strength, and promotion of sleep quality. Future longitudinal research is needed to understand and elaborate on the processes underlying the association between dietary behaviours and depression symptoms.

**Correspondence to:** Chichen Zhang School of Health Management, Southern Medical University No. 1023 Shatai Nan Road, Baiyun District, Guangzhou, Guangdong China zhangchichen@sina.com Yibo Wu School of Public Health, Peking University No. 38 Xueyuan Road, Haidian District, Beijing China bjmuwuyibo@outlook.com
